# Impact of Cone vs. Hetzer Repair on Postoperative Outcomes in Patients With Ebstein's Anomaly: 10-Year Experience From a Single Institution

**DOI:** 10.3389/fcvm.2021.710168

**Published:** 2021-08-05

**Authors:** Qi Lou, Yiping Zou, Jinlin Wu, Jimei Chen, Jian Zhuang, Shusheng Wen

**Affiliations:** ^1^The Second School of Clinical Medicine, Southern Medical University, Guangzhou, China; ^2^Department of Cardiac Surgery, Guangdong Cardiovascular Institute, Guangdong Provincial People's Hospital, Guangdong Academy of Medical Sciences, Guangzhou, China; ^3^College of Medicine, Shantou University, Shantou, China

**Keywords:** Ebstein anomaly, cone repair, hetzer repair, surgery, congenital heart disease

## Abstract

**Objective:** The aim of this study was to compare the early outcomes of the cone and Hetzer procedures for Ebstein's malformation.

**Methods:** This retrospective study included patients who underwent either cone (*n* = 83) or Hetzer repair (*n* = 45) with Ebstein's malformation from January 2011 to December 2020.

**Results:** One early death occurred in the cone group due to low cardiac output syndrome. Five cone and three Hetzer repair patients required reoperation before discharge. At discharge, the cone group had a better reduction in tricuspid valve regurgitation (TR) than the Hetzer group (74.7 vs. 51.1%, *p* = 0.009). Two patients in the cone group and seven patients in the Hetzer group required reoperation >30 days after their initial surgery. The cone group with no/mild TR was 75.6%, and the Hetzer group was 48.9% at the time of last follow-up (*p* = 0.010).

**Conclusion:** Short-term outcomes of the cone repair are better than the Hetzer procedure. The cone repair should be the better option among patients with Ebstein's malformation who need surgical intervention.

## Introduction

Ebstein's anomaly (EA), a rare and complex congenital heart malformation, accounts for <1% of congenital heart defects (1–5). Classical features of patients with EA are the abnormalities of the tricuspid valve (TV) leaflets and right ventricle (RV) (6–8).

The characteristics of EA are the TV downward displacement into the RV, atrialization of RV, and functional status deteriorates of RV (9). Other abnormalities of EA include patent foramen ovale (PFO), atrial septal defect (ASD), ventricular septal defect (VSD), Wolff–Parkinson–White accessory pathway, atrial fibrillation, and myopathy of the RV (8, 10).

Several patients with EA require repair in adulthood (11). TR and right heart failure are the most common reasons for surgical repair. Different surgical interventions are defined due to leaflet hypoplasia and the degree of cardiac malformation. TV repair, TV replacement, ventricular plication, ASD closure, bidirectional cavopulmonary shunt (BCPS), and cryoablation are the most common surgical procedures (12). When technically feasible, repair of the TV is usually preferable to replacement (9, 13, 14). Cone repair and Hetzer repair were the most common types of TV repair in our institution. The Hetzer repair could restore TV functional competence even in severe types in which only remnants of the TV may be valid for valve repair (15). The cone modification is a surgical technique that aims at reducing the severity of TR by constructing a funnel-like valve which is most similar to the physiological TV. Recently, favorable short-term outcomes with the cone repair have been demonstrated by a series of reports (9, 12, 16–18). However, there is no study comparing the specific repair techniques for patients with EA. In the study, we aimed to investigate TV functions, adverse events, and survival outcomes after cone repair of EA and compared the results with Hetzer repair.

## Materials and Methods

### Study Population

Overall, 170 patients older than 1 year with EA who had undergone TV repair surgery at Guangdong Provincial People's Hospital (GDPPH) between January 2011 and December 2020 were retrospectively collected in our study. The exclusion criteria of the study were listed as follows: (1) patients who had undergone TV intervention and (2) patients with other complex heart diseases, including complex conotruncal abnormalities, corrected transposition of the great arteries, pulmonary atresia, and tetralogy of Fallot. In the cone group, 23 patients were Carpentier class A, 37 patients were Carpentier class B, 21 patients were Carpentier class C, and two patients were Carpentier class D. There were 45 patients in the Hetzer group, including 13 patients with Carpentier class A, 17 patients with Carpentier class B, 14 patients with Carpentier class C, and one patient with Carpentier class D. Demographic details and clinical information of the patients we analyzed were age, gender, weight, body surface area (BSA), preoperative TR, left ventricular ejection fraction (LVEF), and baseline New York Heart Association (NYHA) class. During this 10-year study, 45 patients underwent Hetzer repair and 83 patients underwent cone repair were finally included. The median follow-up time for reoperation was 45 (interquartile range 26–63) months, and the median follow-up time for TR was 14 (interquartile range 3–33) months. The study protocol was approved by the Ethics Research Committee of GDPPH. The Ethical committee reference code is KY-Q-2021-106-01. The privacy and personal identification information of the subject were deleted. Thus, the study will not adversely affect the rights of subjects. Patients and patients' legal guardians gave the written consent to store their data anonymously in the hospital database. We obtained their informed consent during hospitalization and contacted the patients or patients' legal guardians *via* phone and WeChat for follow-up. In the preoperative and postoperative examinations, patients were evaluated with transthoracic echocardiography. Patients over 50 years old were evaluated with coronary angiography before the operation.

### Surgical Procedure

The cone technique was developed out of the Carpentier repair (18), which was mainly applied to patients with Carpentier's type A/B or patients with large enough anterior leaflet. Details of the cone procedure were divided into several parts. Firstly, the TV was completely detached from the mid-twelve o'clock point of the surgeon's perspective. Secondly, the chordae at the edge of the valve leaflets were retained, and the mural chordae were cut off. Furthermore, the posterior papillary muscle of RV was detached gradually until it is close to the top of the cone shape. Then, septum and posterior valve leaflets were sutured, which plays the role of chordae and cooperates to form the cone shape. Afterward, the thin and atrialized RV free wall was plicated longitudinally. Meanwhile, like Wu' repair, 5–0 prolene was used to continuously suture atrialized RV transmurally between the coronary arteries. Then, two 3–0 prolenes with buckles were used to reform the true tricuspid annulus, and the size of the true annulus depends on the anterior leaflet. Our institution divides the annulus into four parts and adopts continuous double sutures, then valves and annulus would be stitched together. The annuloplasty ring was usually used to reduce tension when the RV is too large and the new annulus is at risk of tearing.

For the indications of Hetzer repair, this technique is suitable for patients with Carpentier type A/B/C anatomy; those patients need a relatively normal size anterior leaflet and have good activity of the leaflet. If the anterior leaflet activity is restricted, it should be freed from adhesions with the right ventricular free wall to restore the activity of the leaflet. The core principle of this technique for this group of patients is to reduce the anatomic tricuspid ostium so that the most mobile leaflets (anterior leaflet and part of posterior leaflet) may coapt with an opposing structure, septal leaflet, or ventricular septum, during the systole period. Procedures were carried out through median sternotomies with bicaval cannulation and mild to moderate systemic hypothermia. The cardioplegic arrest was initially provided by aortic root infusion of crystalloid arrest solution (crystalloid cardioplegic solution for children and bloody cardioplegia solution for adults). An oblique incision was performed, and the situs was examined. A mattress suture of 3–0 polypropylene pledgeted with autologous pericardium or Dacron patch is passed from the anterior leaflet annulus to the atrialized septum just below the natural septal annulus or the septal anatomic annulus. A small part of these sutures is added toward the posterior annulus. After the sutures are tied, the size of the anatomic tricuspid annulus is decreased. When the saline solution filled the right ventricular cavity, testing valve competence, the anterior part of the anterior leaflet now coapts with the atrialized septum. The atrialized chamber is now incorporated into the contracting right ventricular cavity instead of being plicated. After the Hetzer procedure is finished, the whole TV can be remodeled to act as a valve-closing structure without plication of the atrialized ventricular chamber.

All the patients who underwent the TV replacement were implanted with the biological valve.

### Echo Data

All preoperative and postoperative two-dimensional echocardiograms were measured by cardiologists. The data were obtained from the study within 3 months before surgical intervention, the postoperative at discharge, and 3 months or longer after surgery. Cardiologists evaluated the TV function by transthoracic echocardiography. The TV is usually assessed in the apical four-chamber view. Color Doppler is used to evaluate the degree of TR. The classification of TR is defined as follows: (1) mild TR is defined as the ratio of the length of the regurgitation jet to the area of the right atrium which is <20%, and the length of the regurgitation jet is <1.4 cm. (2) Moderate TR is defined as the ratio which is 20% or more and <40%, and the length of the regurgitation jet is ≥1.4 cm and <3.0 cm. (3) Severe TR is described as the ratio which is more than 40% and the length is more than 3.0 cm.

### Statistical Analysis

Continuous variables were expressed as median and interquartile range and compared by applying the Wilcoxon tests. Categorical variables were summarized as numbers and percentages and compared by using the chi-square test, Yates' correction test, or Fisher's exact test. Survival was estimated by using the Kaplan–Meier survival curves and compared by using the log-rank test.

The statistical analysis of this study was performed using IBM Statistical Package for the Social Sciences version 26.0 (SPSS 26.0, Chicago, IL, USA). The value <0.05 was considered statistically significant in the comparisons. The figures of this study were drawn by R software 4.0.

## Results

### Baseline Patients and Clinical Characteristic

Among the 170 patients with EA who underwent a surgical procedure in our institution, 128 met the inclusion criteria for this study. Of these patients, 83 (64.8%) patients underwent cone and 45 (35.2%) patients underwent Hetzer repair, respectively.

The demographic and baseline characteristics of 128 patients for the two groups are shown in Table 1. More than half of the patients were female (*n* = 89, 69.5%). The majority of patients were older than 18 years (*n* = 85, 66.4%). The severe TR was observed in most patients preoperatively (*n* = 125, 97.7%). The common symptoms included palpitations, syncope, cyanosis, and dyspnea, but most patients were asymptomatic. Generally, patients were in sinus rhythm preoperatively. Rare cases had atrial fibrillation (nine cases), atrial flutter (one case), supraventricular tachycardia (one case), and Wolf–Parkinson–White syndrome (eight cases). One patient had implanted a pacemaker preoperatively. Prior cardiac surgeries were observed in two patients, including surgical ASD closure and intervention ASD closure. A percentage of 18.75% of patients were in preoperative NYHA function class III or IV. The RV diameters between the two groups were also analyzed. There was no statistical difference in the RV diameters preoperatively between the two groups (41 vs. 43 mm, *p* = 0.327).

**Table 1:** Baseline patient demographics and Preoperative characteristics.

**Variables**	**Cone (83)**	**Hetzer (45)**	***p*-value**
Age	27 (7.4–44)	28.9 (14.2–41.35)	0.930
SPO2 (%)	98 (95–99)	97 (95–98.75)	0.393
Weight (kg)	46 (22.5–58)	49 (40.25–60)	0.435
BSA (m^2^)	1.45 (0.88–1.62)	1.47 (1.23–1.66)	0.312
Gender			0.186
Female	61 (73.5)	28 (62.2)	
Male	22 (26.5)	17 (37.8)	
Preoperative palpitation			0.194
No	54 (65.1)	24 (53.3)	
Yes	29 (3.4.9)	21 (46.7)	
Preoperative syncope			1.000
No	79 (95.2)	43 (95.6)	
Yes	4 (4.8)	2 (4.4)	
Baseline NYHA			0.343
I	37 (44.6)	19 (42.2)	
II	28 (33.7)	20 (44.4)	
III	16 (19.3)	4 (8.9)	
IV	2 (2.4)	2 (4.4)	
Atrial fibrillation			1.000
No	77 (92.8)	42 (93.3)	
Yes	6 (7.2)	3 (6.7)	
Atrial flutter			1.000
No	82 (98.8)	45(100.0)	
Yes	1 (1.2)	0 (0.0)	
Supraventricular tachycardia			1.000
No	82 (98.8)	45 (100.0)	
Yes	1 (1.2)	0 (0.0)	
Wolf-Parkinson-White syndrome			0.450
No	79 (95.2)	41 (91.1)	
Yes	4 (4.8)	4 (8.9)	
Cardiothoracic ratio			0.826
Mild (0.4–0.6)	33(39.8)	16 (35.6)	
Moderate (0.6–0.8)	46 (55.4)	26 (57.8)	
Severe (>0.8)	4 (4.8)	3 (6.7)	
Carpentier classification			0.873
A	23 (27.7)	13 (28.9)	
B	37 (44.6)	17 (37.8)	
C	21 (25.3)	14 (31.1)	
D	2 (2.4)	1 (2.2)	
PreTR			0.551
Moderate	3 (3.6)	0 (0)	
Severe	80 (96.4)	45 (100)	
PreLVEF	68.5 (64–74.75)	64 (60–70)	0.005
Previous ASD closure			1.000
No	82 (98.8)	44 (97.8)	
Yes	1(1.2)	1 (2.2)	
Previous ablation			1.000
No	78 (94.0)	42 (93.3)	
Yes	5 (6.0)	3 (6.7)	
Previous pacemaker implantation			1.000
No	82 (98.8)	45 (100.0)	
Yes	1(1.2)	0 (0.0)	
Pre RV diameter	41 (27–52.5)	43 (34–61)	0.327

*BSA, body surface area; NYHA, New York Heart Association; PreTR, pre-operatively tricuspid regurgitation; PreLVEF, pre-operatively left ventricular ejection fraction; Pre RV diameter, pre-operatively right ventricular diameter*.

No statistically significant difference was observed between the two groups except preoperative LVEF. However, the preoperative LVEFs of the two groups were in normal scopes.

### Operative Details and Early Results

Surgical procedure details and TR degree for echocardiography before the discharge of all patients are shown in Table 2. Cardiopulmonary bypass (CPB) time and aortic cross-clamp (ACC) time were significantly higher in the cone group (both *p* < 0.001). However, there was no statistical difference among the two groups for intensive care unit (ICU) staying time (*p* = 0.685) and postoperative hospital staying time (*p* = 0.710). Eight in the cone repair and 10 in Hetzer repair required concomitant BCPS (*p* = 0.051).

**Table 2:** Operative data.

**Variables**	**Cone (83)**	**Hetzer (45)**	***p*-value**
CPBtime	136 (113–179)	90.50 (74.50–122.25)	<0.001
ACCtime	89 (70.50–128.50)	51.00 (35.00–62.25)	<0.001
BCPS			0.051
No	75 (90.4%)	35 (77.8%)	
Yes	8 (9.6%)	10 (22.2%)	
Reoperation before discharge			1.00
No	78	42	
Yes	5	3	
Death			1.00
No	82 (98.8)	45 (100)	
Yes	1 (1.2)	0 (0)	
ICU time	2 (1.00–3.00)	2 (1.00–2.50)	0.685
Hospital time	6 (4.00–9.00)	7 (6.50–9.50)	0.710
Discharge TR			0.009
Mild/No	62 (74.7)	23 (51.1)	
Moderate	11 (13.3)	16 (35.6)	
Severe	10 (12.0)	6 (13.3)	
Discharge LVEF	66 (61–72.5)	67 (62–73)	0.693

*CPB, cardiopulmonary bypass; ACC, aortic cross clamp; BCPS, bidirectional cavopulmonary shunt; ICU, intensive care unit; TR, tricuspid valve regurgitation; LVEF, left ventricular ejection fractions*.

One patient in the cone group died before discharge. A 5-year-old male presented with Carpentier type D, NYHA class III, ASD, and severe TR preoperatively. The preoperative transthoracic echocardiography showed that the right atrium was significantly enlarged with a diameter of 47 mm, and the size of the atrialized RV is 43 ^*^ 37 mm, which is much larger than the functional RV with a size of 15 ^*^ 34 mm. The LVEF of this patient was 65%. The patient had taken cone repair and ASD pert closure. His central venous pressure rose after the chest was closed, combined with unstable blood pressure and functional right ventricular swelling. The surgeon decided to perform BCPS and delayed chest closure to reduce the preload of the RV. The CPB time was 253 min, and the ACC time was 162 min. His symptoms remained after the operation. During the hospital staying time, this patient received reoperation twice due to unstable circulation and poor right heart function on the first and third postoperative days, including ASD enlargement and single ventricular surgery. A large number of vasoactive drugs were needed to maintain blood pressure after surgery. The major cause of in-hospital death was low cardiac output syndrome.

There were five patients (four cases were Carpentier type C, one case was Carpentier type D) who underwent cone repair which required reoperation, and three patients (two cases were Carpentier type B, one case was Carpentier type D) accepted Hetzer repair which needed reoperation. The causes of reoperation were mainly bleeding (one case), excessive regurgitation (four cases), surgical site infection (one case), RV dysfunction (one case), and complete atrioventricular block (one case). Among patients who received reoperation due to excessive regurgitation, three received re-repair (two with Carpentier classification C in cone group, one with Carpentier classification B in Hetzer group) and another one (with Carpentier B in Hetzer group) received the single-ventricle surgery. No statistical difference was found between the two groups in reoperation rate before discharge (*p* = 1.00). The patients who received reoperation before discharge did not receive cardiovascular operation at the follow-up. Only one patient who underwent the single ventricular reoperation in the Hetzer group takes captopril, spironolactone, and aspirin regularly.

In the routine of postoperative echocardiography before discharge, a lower rate of mild/no TR (51.1 vs. 74.7%) and a higher rate of patients with moderate (35.6 vs. 13.3%) and severe (13.3 vs. 12%) TR were observed in the Hetzer group compared with the cone group (*p* = 0.009).

### Follow-Up After Hospital Discharge

**Figure 1:**
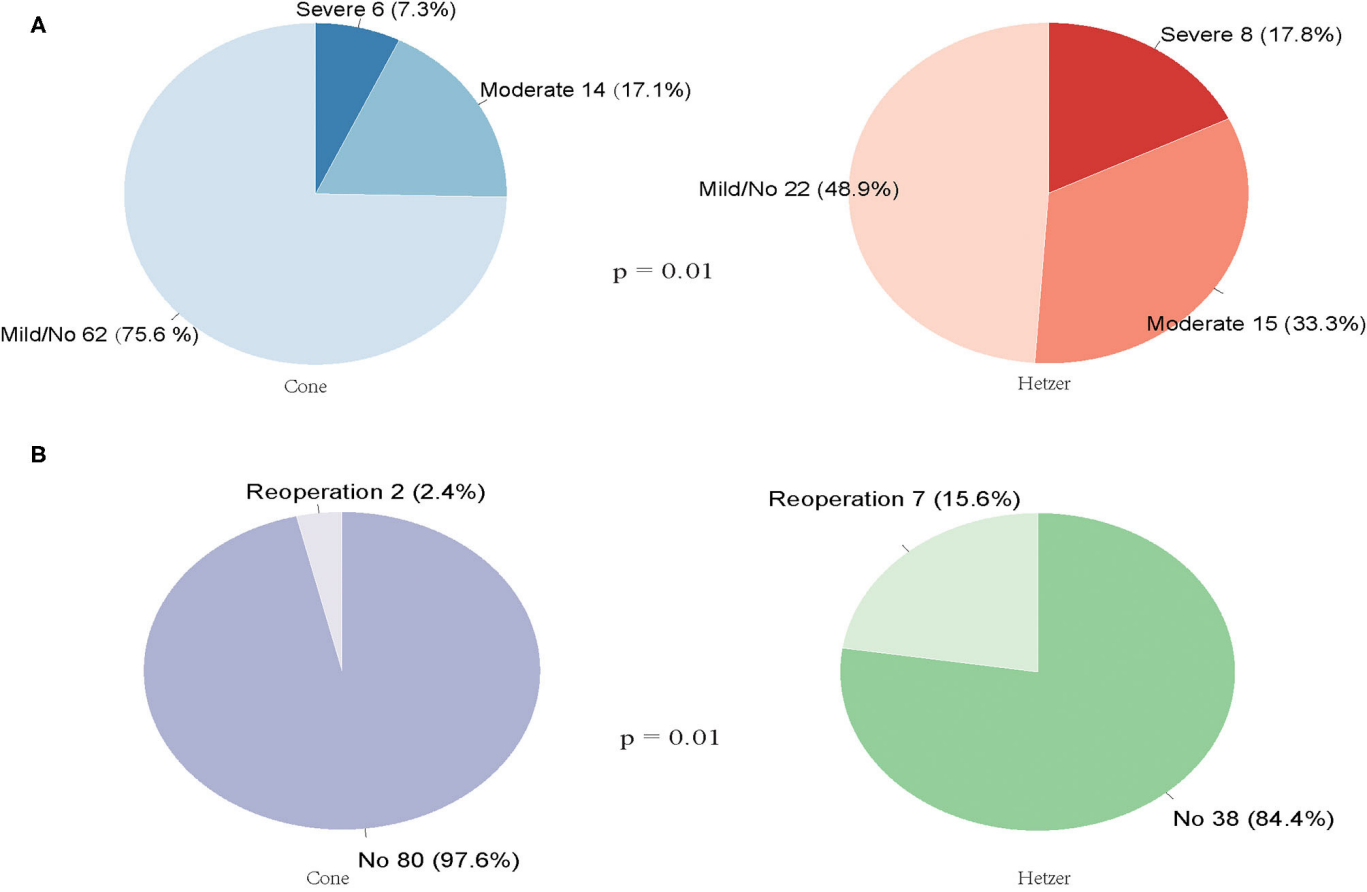
**(A)** The TR of patients after cone or Hetzer repair at 3 months and longer. **(B)** The reoperation rate during the follow-up period of the cone repair and Hetzer repair.

Figure 1A summarizes the TR of patients after surgery at 3 months and longer. The median follow-up time for reoperation was 45 (interquartile range 26–63) months, and the median follow-up time for TR was 14 (interquartile range 3–33) months. The incidence rate of mild or without TR was higher in patients who underwent cone repair than those with Hetzer repair (75.6 vs. 48.9%, *p* = 0.010). In total, nine patients required reoperation during the follow-up period. Seven patients in the Hetzer group required late reoperation due to surgical site infection (one case with Carpentier type A), pericardial effusion (two cases with Carpentier type B), severe TR (two cases with Carpentier type C, one case with Carpentier type B), and arrhythmia (one case with Carpentier type A). Two patients with Carpentier type B in the cone group received reoperation because of arrhythmia (one case) and severe TR (one case). There was a statistical difference between the reoperation rate during the follow-up period of the cone repair and Hetzer repair (2.4 vs. 15.6%, *p* = 0.01) (Figure 1B). Two patients in the Hetzer group received the cone re-repair for severe TR during the follow-up, and another one underwent the TV replacement. Follow-up investigation and analysis were performed on four patients diagnosed with Carpentier type C who underwent the reoperation due to severe TR before discharge or during the follow-up. Two patients in the cone group were both mild or none TR, and one patient in the Hetzer group was mild or none TR; the other one was severe TR.

**Figure 2:**
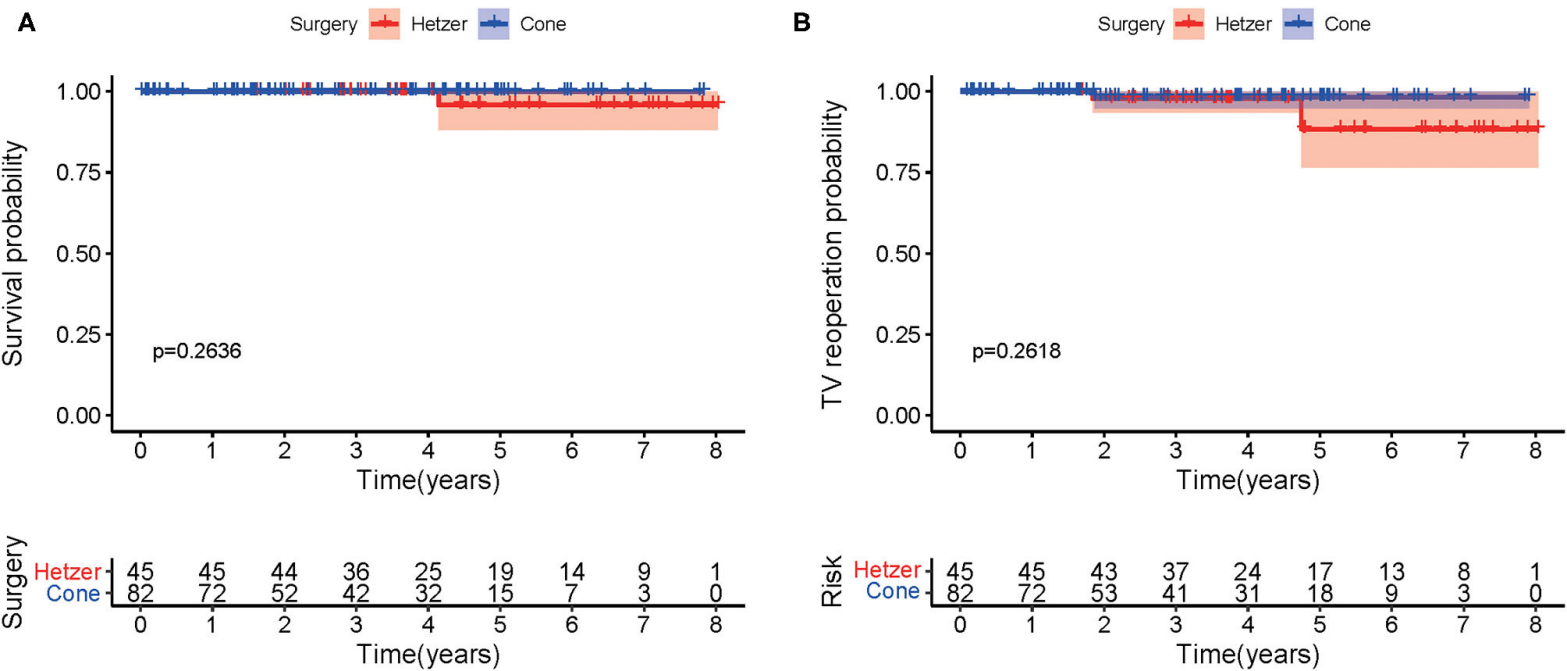
**(A)** The Kaplan–Meier curves for survival after hospital discharge. **(B)** The Kaplan–Meier curve showing freedom from reoperation.

Figure 2A describes the Kaplan–Meier curves for survival after hospital discharge. A comparison with patients with cone repair vs. Hetzer repair revealed no significant difference in overall survival (*p* = 0.2636). One patient who received Hetzer repair had sudden death at 50 months postoperatively. Figure 2B shows the Kaplan–Meier curves for reoperation for recurrent TR between two groups at the follow-up. No significant difference was observed (*p* = 0.2618).

**Figure 3:**
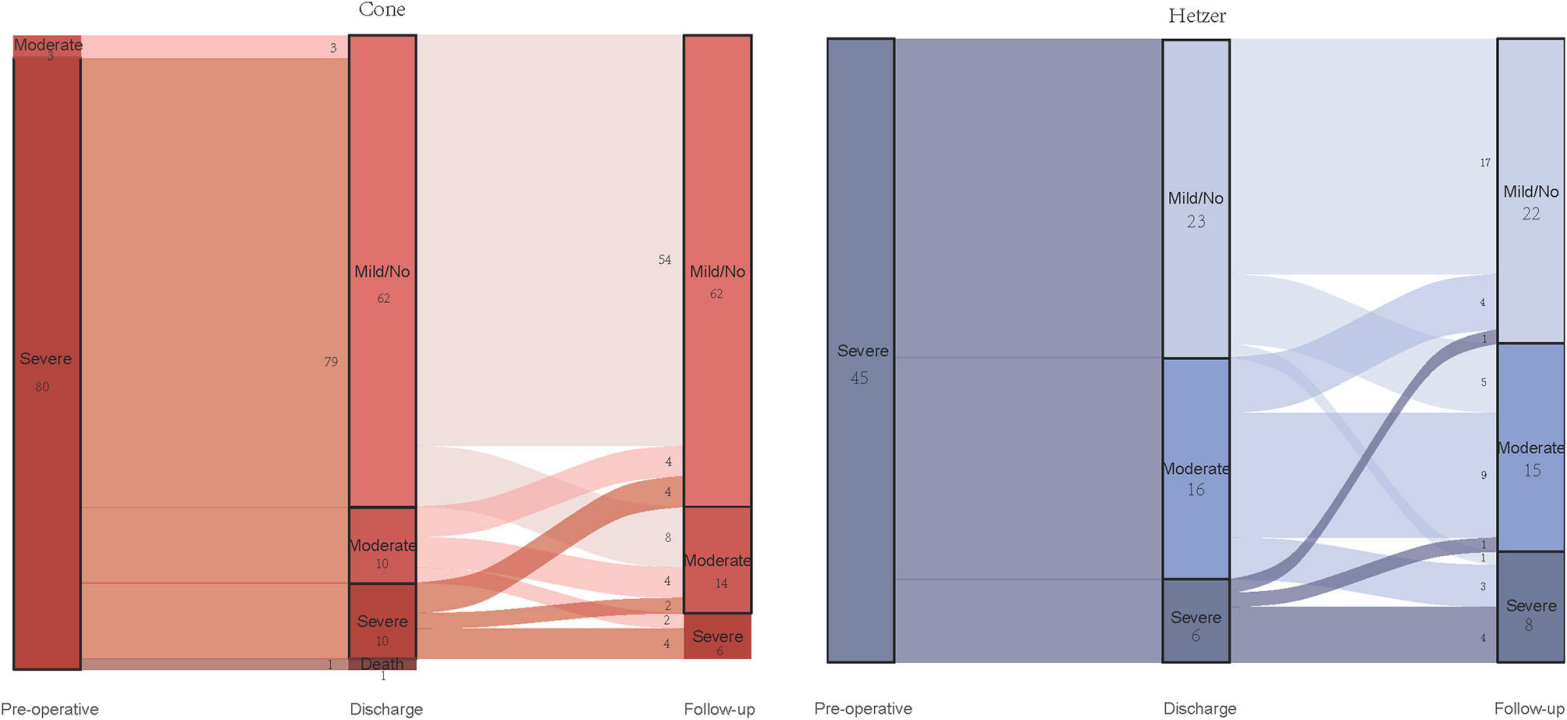
Alluvial diagram showing the changes of the degree of tricuspid valve regurgitation in two groups.

Furthermore, Figure 3 shows the comparison between regurgitation at discharge and last follow-up in two groups. In comparison with the regurgitation at discharge, the follow-up regurgitation of the cone group was improved (*p* < 0.001). Eight patients with mild or no regurgitation progressed to moderate regurgitation after being discharged from the hospital, but no one developed a severe regurgitation. There were 10 cases of severe regurgitation at discharge, four cases reduced to mild or without regurgitation during follow-up time, and two patients relieved to moderate regurgitation. In the Hetzer group, the degree of regurgitation at follow-up was more serious than that at discharge (*p* = 0.001). Five patients with mild or without TR at discharge developed moderate TR, and one patient with mild or without TR developed severe TR. However, in patients with severe TR at discharge, one patient reduced to mild or without TR, and one patient reduced to moderate TR.

There was no statistical difference in the RV diameters at the last follow-up of the two groups (49 vs. 49 mm, *p* = 0.772). Compared with RV diameters before surgery, RV diameters at the last follow-up were significantly increased in the Hetzer group (33 vs. 45 mm, *p* = 0.030). However, although, the trend is also increasing, the RV diameters before operation and those at the last follow-up did not meet statistically significant difference in the cone group (35 vs. 47 mm, *p* = 0.074).

## Discussion

The longitudinal study identified the TV situation and recurrent surgical intervention in patients with EA who underwent cone and Hetzer surgery. Despite that longer CPB time and ACC time were found in the cone repair group, the ICU staying time, and postoperative hospital staying time were similar between the cone and Hetzer groups. It is worth mentioning that the cone repair conferred a significant advantage over Hetzer repair in the TR degree of patients with EA. To the best of our knowledge, this is the first study to assess and compare the TV function after cone and Hetzer repair.

In the surgical treatment of EA, various alternatives depended on cardiac malformation. Hetzer et al. (15) reported the outcome of Hetzer repair for 68 patients with EA. In this study, early and late mortality was reported as 2.9 and 5.8%, respectively. The tricuspid insufficiency of EA patients was significantly improved at 3 months and 1 year after the Hetzer repair (*p* < 0.001), which provided satisfactory long-term outcomes even in severe types of EA. By analyzing the outcomes of 253 patients with EA who underwent cone repair, Holst et al. (19) revealed that cone repair could reduce TR and RV area (both *p* < 0.001).

Recently, Burri et al. (10) reported a comparison of the midterm follow-up period of patients with EA who underwent cone repair and other repairs. They found that the cone repair provided a higher rate of successful repair and a lower rate of moderate/severe TR (*p* = 0.03). Ozbek and his associates (12) reached a similar conclusion that the rate of moderate/severe TR of cone repair was lower than other repairs in patients with EA postoperatively (*p* = 0.009). Vogel et al. (20) found that the cone repair had a higher rate of reduction in TR than conventional surgery in patients with EA (*p* = 0.004). In addition, the longer CPB and ACC time were observed in the cone group than in conventional groups in this study. Our study also demonstrated that cone repair took longer CPB and ACC time but showed an advantage in reduction of TR at discharge and early postoperative outcome compared with Hetzer repair. Compared with the above studies, our research was based on a relatively large cohort that contained patients with EA who underwent two specific surgical procedures. This advantage makes our study more convincing. Being similar to Burri's study, our study also demonstrated that the reoperation rate postoperatively during follow-up was lower in the cone group compared with the Hetzer group. The reason for the better surgical outcome in the cone group might due to the near-normal TV anatomy and function which was provided by cone repair. Unlike the Hetzer group, a leaflet-to-leaflet coaptation rather than a leaflet-to-annulus coaptation was performed in the cone group, which distributes the closing stress to the leaflets uniformly (9). Additionally, researchers demonstrated that the cone repair created greater valvular coaptation by using three leaflets, which could significantly reduce the degree of TR (18). We hypothesize that the reduction of TR would decrease the capacity load of RV and improve the RV function.

The RV volumes after surgery tended to increase compared with those before surgery, but they did not reach statistical significance. This result might be affected by the limited cases and short-term follow-up. Lianza's study (17) indicated that the RV volumes were increased, and the RVEF and FAC of RV were decreased after cone repair. Other reports also showed the decline of RV systolic function after cone repair in a short-term follow-up (21, 22). Lianza believes that the pathological change of the myocardium of RV was the main cause of the continuous deterioration of RV function. Unfortunately, the echo in our institution lacked RV function data, so we did not analyze the RV function in this study.

The decline of RV volume and persistent RV function after the cone repair has been previously noted by several other investigators (16, 23, 24). Beroukhim et al. (16) described the decreased RV volume and the constant RVEF after the cone repair. The lower volume and likely filling pressure of the RV may improve the filling pressure of the left ventricle through the change of septal movement and transmural pressure. According to the Laplace law, the tension of the RV wall was reduced due to the decrease in the RV diameter. Afterward, restoring the entire anatomic RV to the functional portion of the RV may allow more valid stroke volume (19). It was a good sign that the cone repair would prevent a heart failure at follow-up (13).

Our study had several limitations. Firstly, this study is a retrospective research from a single center, which had the potential to introduce selection biases. However, no significant difference was found in the baseline characteristics between cone and Hetzer groups except for preoperative LVEF (both were normal). Secondly, this study assessed postoperative valve function by echocardiographic examination at discharge and short-term outcome but lacked long-term echocardiographic data. Thirdly, the analysis of freedom of reoperation overestimates the function of the TV, including patients with moderate/severe TR but who refuse reoperation. Fourthly, there were fewer preoperative and postoperative two-dimensional echocardiograms that measured the function of the RV. Therefore, this study assessed the short-term results by the reduction of TR alone due to a lack of information on RV function.

## Conclusion

Based on our experience, despite longer CPB and ACC time, cone repair had a higher rate of reduction in TR than Hetzer repair in patients with EA. Compared to Hetzer repair, we prefer cone repair as the treatment of choice for patients with EA.

## Data Availability Statement

The raw data supporting the conclusions of this article will be made available by the authors, without undue reservation.

## Ethics Statement

The studies involving human participants were reviewed and approved by the Ethics Research Committee of Guangdong Provincial People's Hospital. The patients or patients' legal guardians provided written informed consent to participate in this study.

## Author Contributions

QL: collection and analysis of data and manuscript writing. QL and YZ: analysis and interpretation of data. YZ and JW: data acquisition. JC, JZ, and SW: project development and critical revision. All the authors participated in the discussion and editing of the manuscript.

## Conflict of Interest

The authors declare that the research was conducted in the absence of any commercial or financial relationships that could be construed as a potential conflict of interest.

## Publisher's Note

All claims expressed in this article are solely those of the authors and do not necessarily represent those of their affiliated organizations, or those of the publisher, the editors and the reviewers. Any product that may be evaluated in this article, or claim that may be made by its manufacturer, is not guaranteed or endorsed by the publisher.
